# Are caregivers knowledgeable and interested in post-ICU outcomes?

**DOI:** 10.1186/2197-425X-3-S1-A648

**Published:** 2015-10-01

**Authors:** AS Debue, N Kentish-Barnes, G Christine, A Gressent, F Iride, C Bridey, S Calvino-Gunther, S Cosserant, S Renard, C Brossard, E Pichot, B Megarbane, K Klouche, D Flattres-Duchaussoy, C Leroy, C Bigot, B Floccard, JD Chiche, Post-ICU Study Group

**Affiliations:** Hôpital Cochin, Paris, France; Hopital St Louis, Paris, France; CHU Limoges, Limoges, France; CHU Rouen, Rouen, France; Hopital Nord, Marseille, France; CHU Nancy, Nancy, France; CHU Grenoble, Grenoble, France; CHU Clermont Ferrand, Clermont Ferrand, France; CHU Caen, Caen, France; CHU Orléans, Orléans, France; Saint Joseph, Paris, France; Lariboisière, Paris, France; CHU Montpellier, Montpellier, France; CHU Nantes, Nantes, France; CHU Angers, Angers, France; CHU Lyon, Lyon, France; Hopital Cochin, Assitance Publique Hôpitaux de Paris, Paris, France; Paris Descartes University, Paris, France

## Introduction

Long-term outcome after intensive care is a matter of growing interest in the critical care community. As better knowledge of post-ICU outcomes might help improve admission policies, processes of care, or end-of-life decisions, this topic is increasingly covered in medical journals. Access to this information may still be limited for ICU caregivers who play a pivotal role in the delivery of intensive care as well as in decision-making that lead to limitation of care. Whether ICU caregivers have a fair knowledge of long-term outcomes of ICU patients is unknown.

## Objectives

Assess what is the knowledge of ICU caregivers related to long-term ICU outcomes.

## Methods

This study was performed in 19 ICUs in France (12 MICUs, 5 mixed ICUs & 2 SICUs). We designed and conducted a 42-item survey composed of 3 parts: i) demographic data to assess the profile of the caregivers, ii) caregiver's knowledge of outcome data and possible long-term outcomes for ICU patients, iii) caregivers's knowledge of discharge procedures in their ICU. The survey was distributed to all nurses, aid-nurses, chief-nurses and physiotherapists with a goal to collect at least 25 replies/centre. A specific questionnaire was filled by a senior investigator in each centre to collect centre characteristics and outcome data.

## Results

The survey was completed by 445 caregivers (32 [IQ 27-39] y.o.; 80 %female) working in 19 ICUs as nurses (65.2%), aid-nurses (23.6%), chief-nurses (4%), physiotherapists (3.8%) or psychologists (0.4%). Whereas 89% declare that they are interested in post-ICU outcomes, 84% claim they rarely obtain information about patients outcome after ICU discharge and 74% wish they could « systematically obtain news from discharged patients ». Most caregivers claim that they mainly seek feedback from patients with prolonged ICU stays (74%), patients with whom they had a privileged relationship (66%), who had a striking personal history (59%), or from young patients (51%). Patients and their families are the main source of information regarding post-ICU outcomes through letters (71%) or occasional visits (72%). Still, 83% admit to have only rare occasions to meet with former patients. When meeting with former patients, the 2 main topics discussed with caregivers are ICU memories (74%) and QOL (72%), whereas less than 40% discuss physiological and psychological sequels.

## Conclusions

Despite high-interest in understanding long-term outcomes of their patients, caregivers have a limited knowledge of what happens after ICU discharge and most ICUs have no systematic approach to collect long-term ICU outcomes. Creation of multidisciplinary post-ICU clinics could help caregivers to better understand the burden of post ICU sequels.
